# Bias evaluation and reduction in 3D OP-OSEM reconstruction in dynamic equilibrium PET studies with ^11^C-labeled for binding potential analysis

**DOI:** 10.1371/journal.pone.0245580

**Published:** 2021-01-22

**Authors:** Cláudia Régio Brambilla, Jürgen Scheins, Ahlam Issa, Lutz Tellmann, Hans Herzog, Elena Rota Kops, N. Jon Shah, Irene Neuner, Christoph W. Lerche

**Affiliations:** 1 Institute of Neuroscience and Medicine, INM-4, Forschungszentrum Jülich GmbH, Jülich, Germany; 2 Department of Psychiatry, Psychotherapy and Psychosomatics, RWTH Aachen University, Aachen, Germany; 3 Institute of Neuroscience and Medicine, INM-11, Forschungszentrum Jülich GmbH, Jülich, Germany; 4 JARA–BRAIN–Translational Medicine, RWTH Aachen University, Aachen, Germany; 5 Department of Neurology, RWTH Aachen University, Aachen, Germany; Fondazione Istituto G.Giglio di Cefalu, ITALY

## Abstract

Iterative image reconstruction is widely used in positron emission tomography. However, it is known to contribute to quantitation bias and is particularly pronounced during dynamic studies with ^11^C-labeled radiotracers where count rates become low towards the end of the acquisition. As the strength of the quantitation bias depends on the counts in the reconstructed frame, it can differ from frame to frame of the acquisition. This is especially relevant in the case of neuro-receptor studies with simultaneous PET/MR when a bolus-infusion protocol is applied to allow the comparison of pre- and post-task effects. Here, count dependent changes in quantitation bias may interfere with task changes. We evaluated the impact of different framing schemes on quantitation bias and its propagation into binding potential (BP) using a phantom decay study with ^11^C and 3D OP-OSEM. Further, we propose a framing scheme that keeps the true counts per frame constant over the acquisition time as constant framing schemes and conventional increasing framing schemes are unlikely to achieve stable bias values during the acquisition time range. For a constant framing scheme with 5 minutes frames, the BP bias was 7.13±2.01% (10.8% to 3.8%) compared to 5.63±2.85% (7.8% to 4.0%) for conventional increasing framing schemes. Using the proposed constant true counts framing scheme, a stabilization of the BP bias was achieved at 2.56±3.92% (3.5% to 1.7%). The change in BP bias was further studied by evaluating the linear slope during the acquisition time interval. The lowest slope values were observed in the constant true counts framing scheme. The constant true counts framing scheme was effective for BP bias stabilization at relevant activity and time ranges. The mean BP bias under these conditions was 2.56±3.92%, which represents the lower limit for the detection of changes in BP during equilibrium and is especially important in the case of cognitive tasks where the expected changes are low.

## Introduction

Iterative image reconstruction algorithms based on the known maximum likelihood—expectation maximization (ML-EM) have been widely used in positron emission tomography (PET) over the last three decades, with the ordered-subset (OS) variants being particularly prevalent [1–4]. However, these methods have been shown to cause quantitation bias for applications involving low count levels [5–9]. This effect is often problematic in dynamic PET studies with ^11^C-labeled radioligands, which frequently present low counts per frame towards the end of the acquisition interval. Other groups using ML-EM based reconstruction methods have reported different levels of bias [7] and overestimation, as well as underestimation in volumes of interest (VOIs) with either low or high activity concentrations [10]. This bias is particularly relevant in the case of neuro-receptor binding studies that use reference regions, e.g., cerebellum or pons/brainstem, which frequently present low neuro-receptor concentration levels and thus low coincidence counts. In the case of 3D OSEM based reconstruction algorithms, the source of the bias has been attributed to the introduction of a positive bias in the reconstructed images due to the non-negativity constraint in the data prior to correction [11]. To avoid bias from the non-negativity constraint, 3D ordinary Poisson OSEM (OP-OSEM) can be used as an alternative iterative reconstruction method as it implements all required data corrections in a way that preserves non-negativity during the reconstruction [6, 12]. However, 3D OP-OSEM uses observed random and scattered coincidence events without updating them for each iteration step during the image reconstruction [13]. The accuracy of this method has been studied and a bias of 10% or more was reported [11] in regions of a homogeneous phantom. Byars and colleagues [14] showed that the bias can be reduced when a variance reduction algorithm is implemented to reduce the variance in estimated random counts (VRR). Nonetheless, it is important to consider that other factors can also contribute to the quantitation bias in image reconstruction at low count rates, including scatter correction implementations (e.g., frame-based), the framing scheme, and convergence [10, 15, 16]. Moreover, the MLEM and OP-OSEM reconstructions tend to be biased in regions with low activity concentrations and converge much more slowly, meaning that a very high number of iterations would be required to eliminate the positive bias [16]. In addition, different VOIs can differ in convergence according to their shape, volume, and activity concentration; thus, convergence is achievable at a certain number of iterations, but some regions may still converge faster than others. To date, new reconstruction methods had been proposed where regularized algorithms are implemented and improvements in image quality due to noise reduction and improved spatial resolution can be observed [17–19]. However, to the best of our knowledge, these studies did not evaluate the count dependent changes of the quantitation bias for low counts during dynamic PET acquisition studies, or its propagation into binding potential (BP) values.

The aim of this study was to evaluate the impact of different framing schemes by using 3D OP-OSEM reconstruction to assess the quantitative accuracy of PET images. This was achieved with an ^11^C filled phantom decay study, where the evaluated conventional framing schemes have been previously applied in bolus plus infusion (BI) and bolus protocols with [^11^C]ABP688 (ABP, a glutamatergic receptor ligand) [20–25]. Particular attention was given to bias effects on the values of BP at count rate levels usually found at equilibrium in dynamic ABP PET studies. In addition, an alternative method for gathering the PET coincidences into a framing scheme is also proposed in which the true coincidence counts per frame are kept at the same value for all reconstructed frames of the dynamic acquisition. As anticipated in [12, 16], the bias depends partly on the number of counts in the frame. This leads to the assumption that the BP bias values will remain constant throughout the entire acquisition range when the true coincidence counts per frame is consistently maintained over the entire time interval of the dynamic PET acquisition. Based on this premise, different framing schemes were compared with respect to quantitation BP bias and its variation at count rate levels equivalent to the ABP equilibrium phase of the acquisition time range. This evaluation also enables the identification of the lower limit of the BP bias range that a task must overcome in order to be effectively identified (i.e. how much change a task must induce to exceed the bias limits). A spherical and a background VOI (from an adapted NEMA phantom mimicking high and low neuro-receptor density regions) [26] were analyzed using our BI protocol with ABP to evaluate the limitations in binding quantification due to the bias.

## Materials and methods

### PET phantom acquisition

The PET data were acquired using a 3T hybrid MR-BrainPET insert system (SIEMENS, Erlangen, Germany) in list mode [27]. The coincidences were corrected for random events using the delayed window technique with VRR, dead time, attenuation and scattered coincidences (single scatter simulation–SSS method), and physical decay. The image reconstruction was performed with the vendor-supplied 3D OP-OSEM [28] with 2 subsets and 32 iterations (which is the default setting in our institution), and an isotropic voxel size of 1.25 mm into a volume consisting of 153 transverse slices of 256 × 256 pixels. The available 3D OP-OSEM does not include any kinds of penalized likelihood algorithms or point spread function methods for resolution improvements or noise suppression. Post-processing was performed with a 2.5 mm 3D Gaussian filter. Pmod software 3.9 was used to define the VOIs and to extract the activity concentration (kBq/cm^3^) for the analysis.

An adapted NEMA phantom [29], without lung insert and with six sphere inserts, was used. One insert was filled with ^11^C and the others contained cold (no activity) water contrasting in a warm background cylinder. Coincidence data were acquired during eight isotope half-lives, giving 163 minutes total acquisition time for ^11^C, which has a half-life of T_1/2_ = 20.35 minutes. An interval of three T_1/2_ (3T1/2—referred to as T4, T5 and T6), from 61 minutes to 122 minutes of the acquisition time, was used for data analysis. This acquisition time interval was chosen based on the typical count rates measured in our human brain study with ABP, a BI protocol [26] and a scan time of 65 minutes (3T_1/2_ of ^11^C). The ratio of the activity concentration between the sphere (Hot with 28 mm of nominal internal diameter) and the background region (REF) was 1.85:1. This value is frequently found for the ratio of activity concentrations in the grey matter cortex (GM) and cerebellar grey matter (CER, reference region) during the steady-state condition in ABP studies. The activity concentration for the time intervals T4, T5 and T6 during the ^11^C decay study started at T4 with REF containing 8.08 kBq/cm^3^ and 14.99 kBq/cm^3^ in the Hot sphere.

A cold transmission scan of the adapted NEMA phantom using ^68^Ge sources was acquired in a Siemens ECAT Exact HR+ PET scanner. This acquisition (2 bed positions, 20 minutes of transmission, reconstructed with OSEM 2D – 6 iterations and 16 subsets and a 256 × 256 matrix) was used to create the attenuation map for the phantom used in the ^11^C decay study.

**Fig 1:**
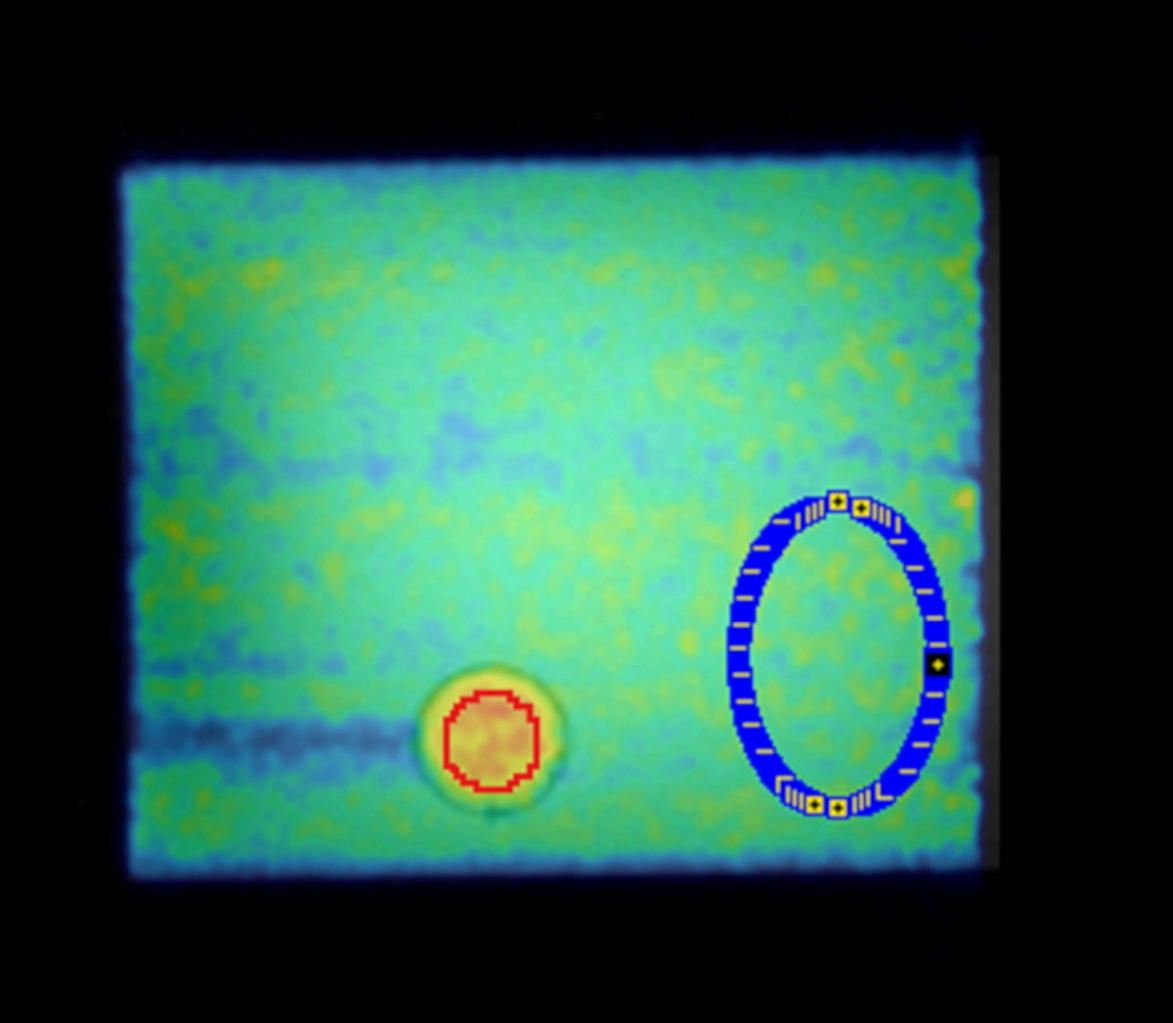
Sagittal PET image from the adapted NEMA phantom overlaid with an MR T1 image during the ^11^C filled phantom decay study. The high activity concentration region (red sphere VOI) and low activity concentration region (blue VOI) were used for bias analysis. The remaining spheres were not included in the study.

To obtain the ground truth, the activity concentrations in the two phantom compartments were measured with a gamma counter (WIZARD automatic gamma counter–PerkinElmer^®^) repeating for 3 probes in a solution of 0.5 ml. Decay correction, the counter calibration factor and volumes/weights of the probes were considered and applied. Fig 1 shows a sagittal PET image from the adapted NEMA phantom overlaid with an MR T1 image and the drawn VOIs.

Based on the known neuro-receptor distribution of ABP [30], the background VOI (REF) was considered to be representative of CER as a reference region and the hot sphere VOI (Hot) was considered to be representative of GM regions, i.e., parts of the cingulate cortex (a neuro-receptor rich region in ABP). The effective volumes of the two VOIs were: 122.32 ml (REF, 62635 voxels) and 4.17 ml (Hot, 2119 voxels), respectively. To reduce partial volume effects in the analysis, the Hot VOI was drawn with a distance of 4 mm to the sphere’s inner borders (internal diameter of 19.97 mm). After applying all data corrections, a constant activity concentration should be observed for the entire acquisition time (T1 to T8) in all phantom compartments of the ^11^C filled phantom. This mimics the constant radioligand concentrations in the brain compartments with an activity ratio as observed in the BI protocol with ABP.

### Reconstruction–frame schemes

**Fig 2:**
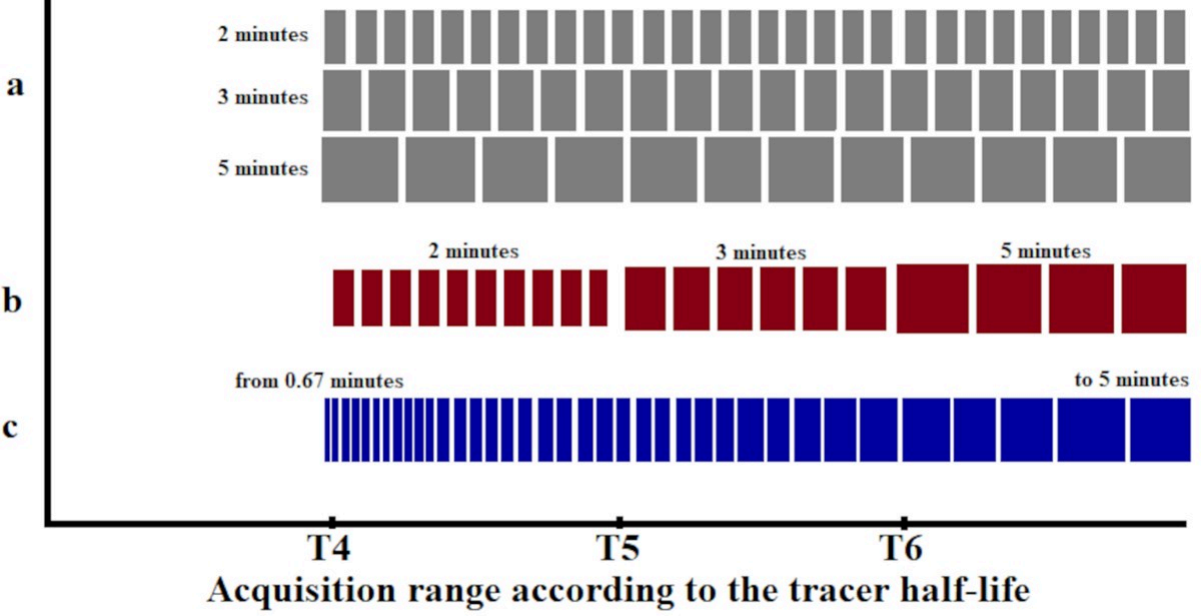
Schematic and chronological representation of the time framings as a function of ^11^C half-life for each framing scheme. **a** are schemes with constant frame length, **b** represents a conventionally increasing frame length scheme and **c** is the increasing frame length scheme with constant trues counts.

The different framing schemes used to evaluate the bias in the time-activity curves (TACs) obtained from the reconstructed images and in the BP values were defined by either constant or increasing frame lengths (still with varying counts) and by an alternative framing scheme with variable frame lengths but constant true coincidence counts that takes the decreasing count rate during the dynamic PET into account. The framing schemes were defined as follows and are represented schematically in Fig 2.

#### Constant frame length schemes (Const)

PET list-mode data of the entire acquisition were sorted into time frames with constant frame lengths of 2, 3 or 5 minutes, respectively (Const 2 min, Const 3 min and Const 5 min). The Const 5 min frame also represents similar time framing to previous studies with ABP [20, 22, 23, 30]. The Const 2 and Cont 3 min framing schemes were included to observe the bias for a count rate per frame range (from 3.7×10^7^ to 3×10^6^) comparable to other studies [7, 10, 28, 31].

#### Conventional increasing frame length scheme (Incr)

PET list-mode data were sorted into time frames with increasing frame length, i.e., during T4 the frame length was set to 2 minutes and during T5 and T6 the frame length was set to 3 minutes and 5 minutes (Incr 2-3-5 min), respectively. In this way, the lower counts for later frames caused by the radioactive decay of ^11^C was compensated to some extent, however, the motivation for this scheme is the adequate sampling of the TAC and not a constant quantification bias. Similar time framing was applied in previous studies with ABP [24, 25].

#### Increasing frame length scheme with constant true coincidence counts (Const Trues)

Total true coincidence rates versus time curves were extracted from the acquisition without image reconstruction and the frame lengths were adjusted to values which yielded the same total counts per frame for all frames of the dynamic PET data. The number of counts in the final frame of the acquisition was taken as a reference for counts per frame. Earlier frames were accordingly shorter. A duration of 5 minutes was chosen for the final frame since this is a typical setting for applications with ketamine tasks [25, 32] and provides sufficient compatibility with our cognitive task-time interval defined by MR in simultaneous PET/MR applications with our BI protocol.

### Bias analysis

Bias and bias variability were analyzed as follows:

#### Activity concentration accuracy (bias)

The bias of the measured activity concentration (A_measured_) was computed by:

$$\mathrm{Bias}\;[\%]=\left[\frac{\left(A_{measured}-A_{ground\_truth}\right)}{A_{ground\_truth}}\right]\times 100$$ (1)

where A_ground_truth_ is the true activity concentration from the probes measured in the gamma counter (ground-truth value) and A_measured_ is the mean activity concentration in the REF (blue VOI in Fig 1) or Hot (red VOI Fig 1) in each frame. Box plots were used to represent the percentage bias variability of the measurement for each VOI per frame. The percentage bias for activity concentration values were computed for all reconstruction frames in each of the different acquisition intervals T4, T5, T6 (half-lives), according to the applied framing scheme described in the Reconstruction–Frame schemes section. Thus, the percentage bias estimated in several frames per half-life allows to evaluate the bias distribution per acquisition interval.

#### Binding potential accuracy (BP bias)

The procedure described in (1) was also used to estimate the bias and bias variability of the BP values by considering BP instead of the activity concentration A. The BP_ground truth_ for the Hot VOI region was estimated as presented in (2). Eq 3 was used to calculate BP_measured_.

$${\mathrm{BP}}_{\mathrm{ground}\;\mathrm{truth}}=\left[\frac{A_{ground\_truth\_Hot}}{A_{ground\_truth\_REF}}\right]‐1$$ (2)

$${\mathrm{BP}}_{m\mathrm{easured}}=\left[\frac{A_{measured\_Hot}}{A_{measured\_REF}}\right]‐1$$ (3)

where A_measured_Hot_ is the mean activity concentration in the Hot VOI (high activity concentration region) and A_measured_REF_ is the mean activity concentration in the REF VOI (low activity concentration region). Box plots were used to represent the percentage BP bias variability of the measurement for each frame. The percentage BP bias values were computed for all reconstruction frames in each of the different acquisition intervals T4, T5, T6 (half-lives), according to the applied framing scheme described in Reconstruction–Frame schemes section. Thus, the percentage BP bias estimated in several frames per half-life allows to evaluate the BP bias distribution per acquisition interval. The BP values used in the study are based on the simple ratio methods [33].

#### Analysis of the relative bias changes

This analysis was performed to evaluate how bias change per time interval and between the intervals (T4, T5 and T6) in the acquisition. Based on this evaluation, TACs recorded in the phantom were also analyzed by evaluating the slope of a straight line fitted to the activity concentration and to the BP values over acquisition time. The percentage change per hour (%/h) of the TACs and BP values was evaluated for the acquisition intervals (T4, T5 and T6) and compared. By choosing the Const Trues framing scheme, we hypothesized that the same bias (closer bias values between acquisition intervals) would be obtained throughout the BP values during the entire acquisition time range (T4, T5 and T6). Thus, the bias should not significantly change the slope of the straight line fitted to the BP values versus time. However, bias from framing schemes that do not consider constant true coincidence counts per frame, such as Const or Incr schemes, may show different slope values between the time intervals (higher slopes and differences between acquisition intervals).

#### Standard error (SE)

Statistical SE was computed according to (4) and Gaussian error propagation was used to estimate the SE for the BP values as SE_BP_ in (5) for the relative bias changes analysis evaluating the slope of the TACs during the time intervals.

$$\mathrm{SE}=\left[\frac{\sigma }{\sqrt{n}}\right]$$ (4)

$${\mathrm{SE}}_{\mathrm{BP}}=\sqrt{{SE}_{Hot}^{2}+{SE}_{REF}^{2}}$$ (5)

where σ is the standard deviation in the VOI regions and *n* is the number of pixels in the VOI.

The TACs represent either the mean activity concentration or the mean BP values per frame versus time during the entire acquisition. For analyzing the slope of these TACs, SE values have been used as error bars and for error progression SE_BP_.

This study was mainly performed with a phantom. We repeated the reconstructions methods presented in Reconstruction–Frame schemes section for a human subject data set example and analyzed through the percentage change per hour (%/h) described in Analysis of the relative bias changes section (results are reported in S1 File and in the discussion section). This study was part of an ongoing study approved by the Ethics Committee of the Medical Faculty of the RWTH Aachen University, Germany and the German Federal Office for Radiation Protection (Bundesamt für Strahlenschutz). No additional activity was injected in the subject (healthy man adult) for the purpose of the report presented here. Written informed consent was obtained from the subject and the study was conducted according to the principles expressed in the Declaration of Helsinki.

## Results

### Activity concentration accuracy

**Fig 3:**
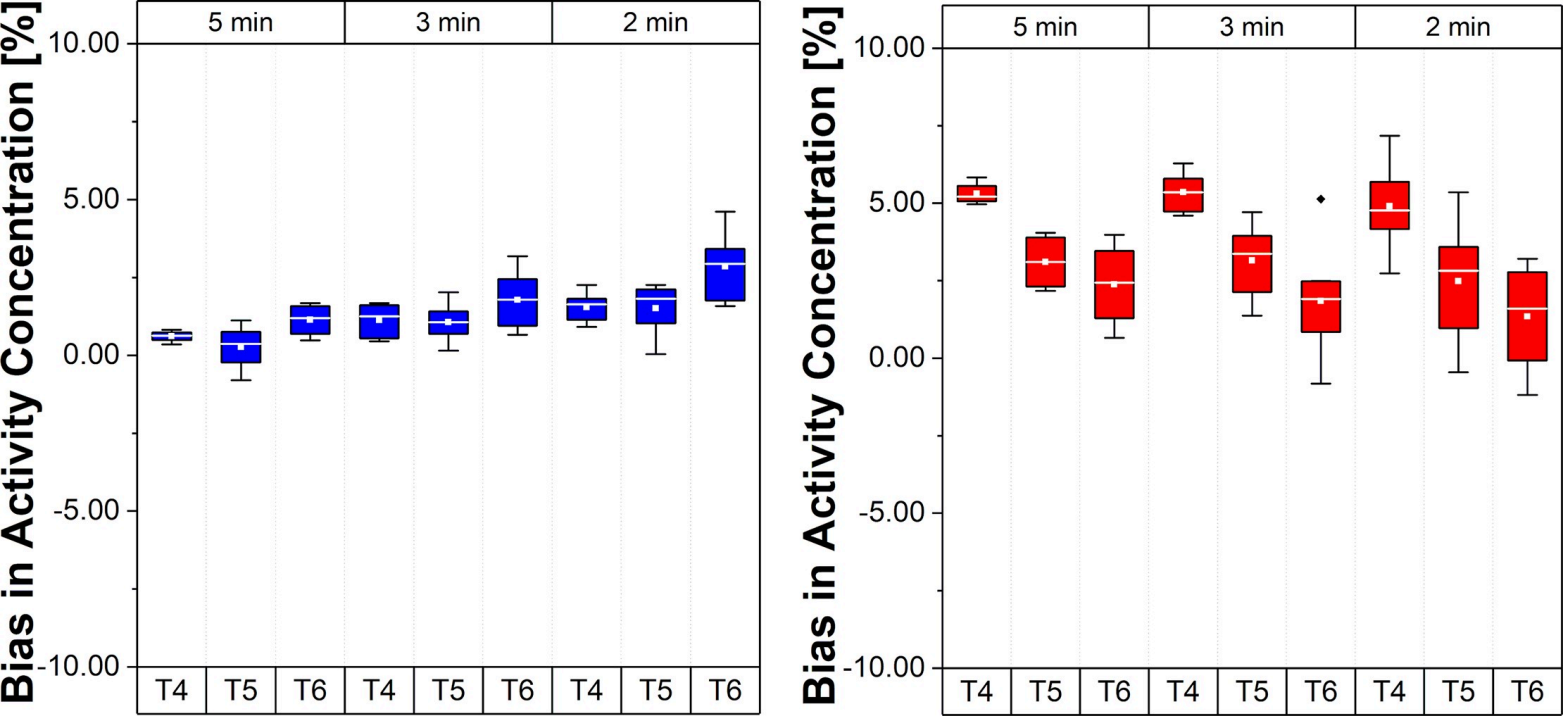
Bias and bias variability in activity concentration for different constant framing schemes. (A): REF VOI region (low activity concentration). (B) Hot VOI region (high activity concentration). Values in the plots are % bias per VOI region, per frame, per time interval and per framing scheme; whiskers show the maximum and minimum, the mean is represented by solid white squares and the median is represented by the white lines; box is 25%-75% and outliers are represented by black points.

Fig 3 shows the relative bias for the two regions in the phantom, for the intervals T4, T5, and T6, and for the three different Const framing schemes explained in Reconstruction–Frame schemes section.

**Fig 4:**
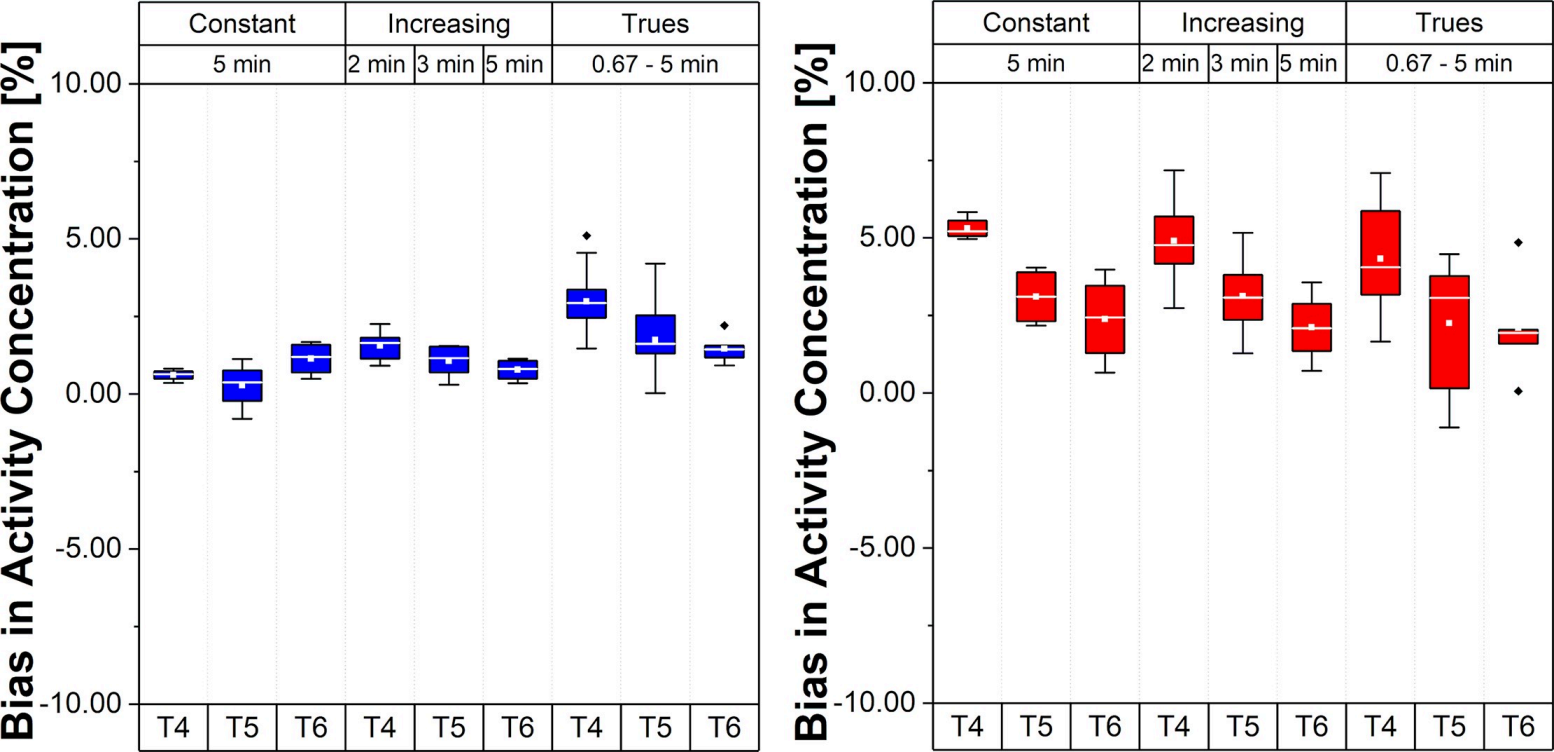
Bias and bias variability in activity concentration for different framing schemes. (A) REF VOI region (low activity concentration region). (B) Hot VOI region (high activity concentration region). Values in the plots are % bias per VOI region, per frame, per time interval and per framing scheme; whiskers show the maximum and minimum, the mean is represented by solid white squares and the median is represented by the white lines; box is 25%-75% and outliers are represented by black points.

In Fig 3A it is possible to notice a slightly increased bias, changing from close to zero towards positive mean values, in the REF VOI region for decreasing frame lengths, i.e. lower count statistics per frame, especially in the T6 interval. In contrast, a different trend is observed in the Hot VOI region where the bias becomes smaller from T4 to T6. With decreasing frame length, the higher positive mean bias values are closer to zero. Fig 4 compares the bias for the two phantom regions, for the three intervals T4, T5, and T6, and for the Const 5 min framing scheme, the Incr and Const Trues schemes described in Reconstruction–Frame schemes section.

A similar trend (as observed in Fig 3A) for the REF VOI region (Fig 4A) can be noticed in terms of a higher mean bias with decreasing frame length if the T4 is compared between different framing schemes (this is as expected). However, a reduced bias in T6 and an opposite trend from T4 to T6 can be observed for Incr and Const Trues schemes. In the Hot VOI region (Fig 4B), the bias trend is maintained, but there is a reduced bias variability in the T6 time interval for Incr and Const Trues schemes compared to Const schemes. This is particularly noticeable with the Const Trues scheme.

### Binding potential accuracy

**Fig 5:**
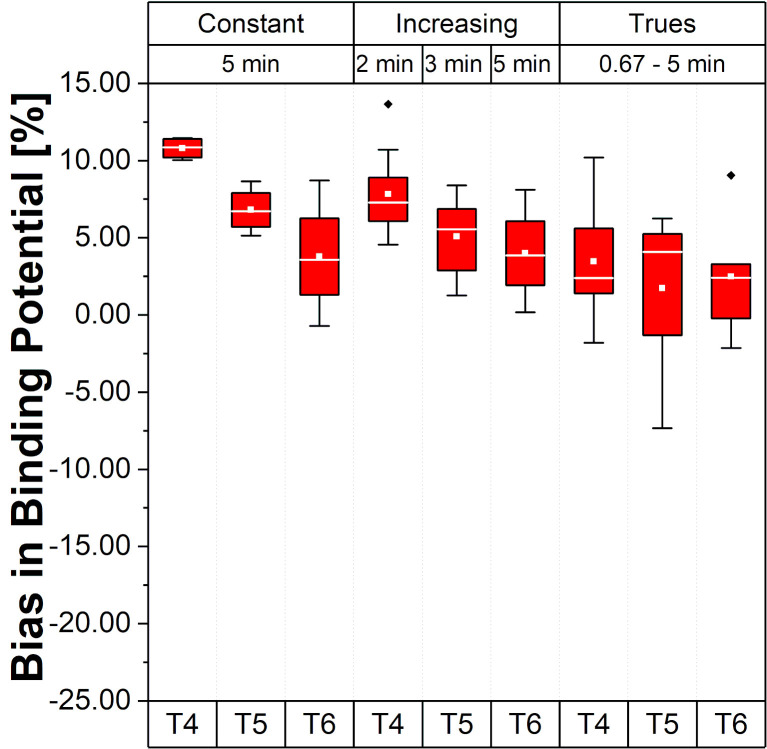
Bias and bias variability of BP values for the Hot VOI region. Values in the plot are % bias in BP, per frame, per time interval and per framing scheme; whiskers bars show the maximum and minimum; the mean is represented by solid white squares and the median is represented by the white lines; box is 25%-75% and outliers are represented by black points.

Fig 5 presents the relative errors of BP values in the Hot VOI region for the different framing schemes and time intervals.

Note that, for the scheme with Const 5 min framing, the bias varies from 10.8% to 3.8% (mean BP bias 7.13±2.01%), suggesting a different bias range according to the time interval (T4, T5 and T6) during the acquisition varying around 7%. These changes were smaller for Incr with a BP bias range from 7.8% to 4.0% (mean BP bias 5.63±2.85%). For the Const Trues framing scheme, the BP bias became smaller than 5% (mean BP bias 2.56±3.92%) with a range from 3.5% to 1.7%.

### Analysis of the relative bias changes

**Fig 6:**
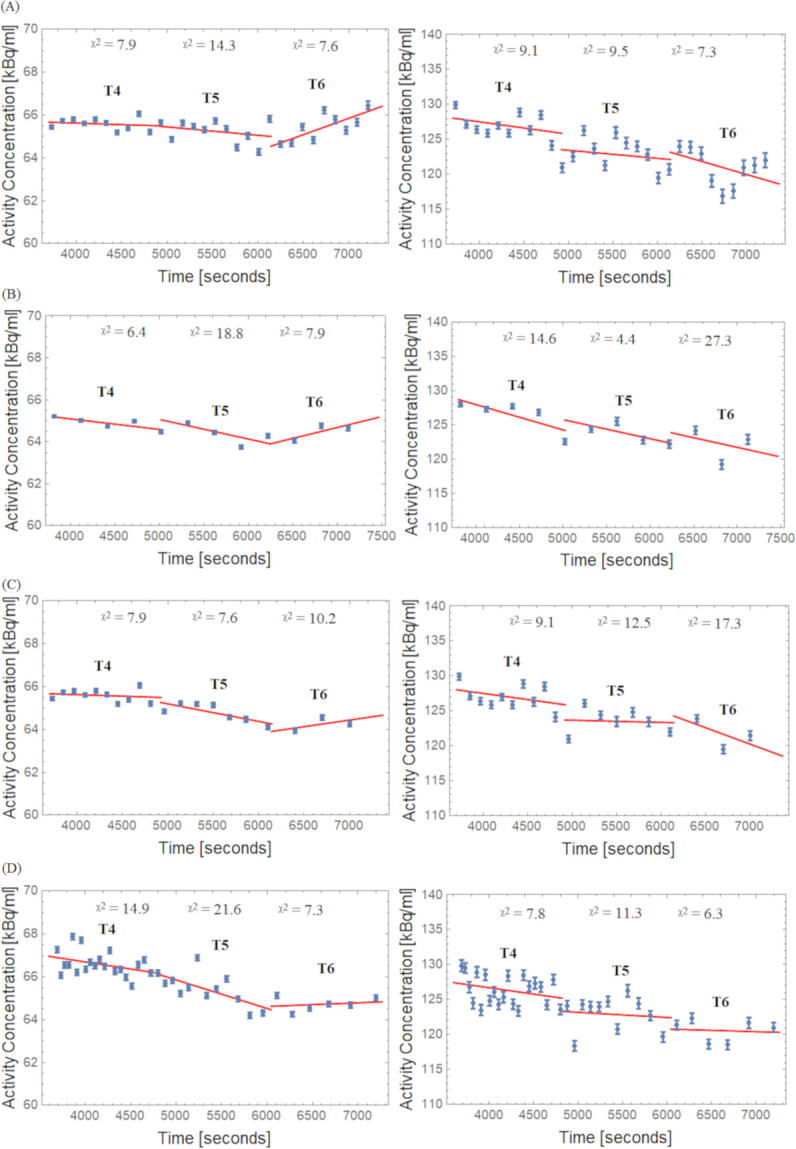
**TACs and linear fits for the REF (left side) and Hot (right side) VOI regions.** (A) Const 2min, (B) Const 5min, (C) Incr and (D) Const Trues framing schemes; X ^2^: Goodness of linear fit over degrees of freedom.

As shown in Fig 6 for four different framing schemes, the TACs of VOIs (REF and Hot) were approximated separately by individual linear fits for the intervals T4, T5 and T6. Looking at the REF region, a similar trend as found in Fig 6 can be seen in the box plots (Figs 3 and 4), which show a negative slope in T4 and T5 and a positive slope in T6 (more pronounced in Const schemes). Note the negative slopes in the Hot VOI region for the different framing scheme methods. Slope values ± uncertainty for the decay corrected TACs are outlined in Table 1.

**Table 1:** Slope* values for time-activity curves.

Frame Scheme	Half-Life (T_1/2_) Interval	Slope (%/h) in REF	Slope (%/h) in Hot
Const 2 min	T4	-0.8 ± 1.4	-5.0 ± 4.3
	T5	-2.1 ± 2.2	-3.4 ± 5.2
	T6	8.4 ± 2.7	-11.1 ± 8.1
Const 5 min	T4	-2.8 ± 1.0	-10.5 ± 4.3
	T5	-5.2 ± 3.3	-8.1 ± 4.6
	T6	5.8 ± 4.4	-8.3 ± 23.2
Incr	T4	-0.8 ± 1.4	-5.0 ± 4.3
	T5	-4.5 ± 1.6	-0.9 ± 5.7
	T6	3.5 ± 4.8	-13.9 ± 17.7
Const Trues	T4	-3.6 ± 1.8	-5.2 ± 3.9
	T5	-7.3 ± 3.0	-2.2 ± 6.4
	T6	0.9 ± 2.1	-4.4 ± 8.0

*Slope–Obtained from linear fits for data presented in Fig 6, and corresponding ± uncertainty.

**Fig 7:**
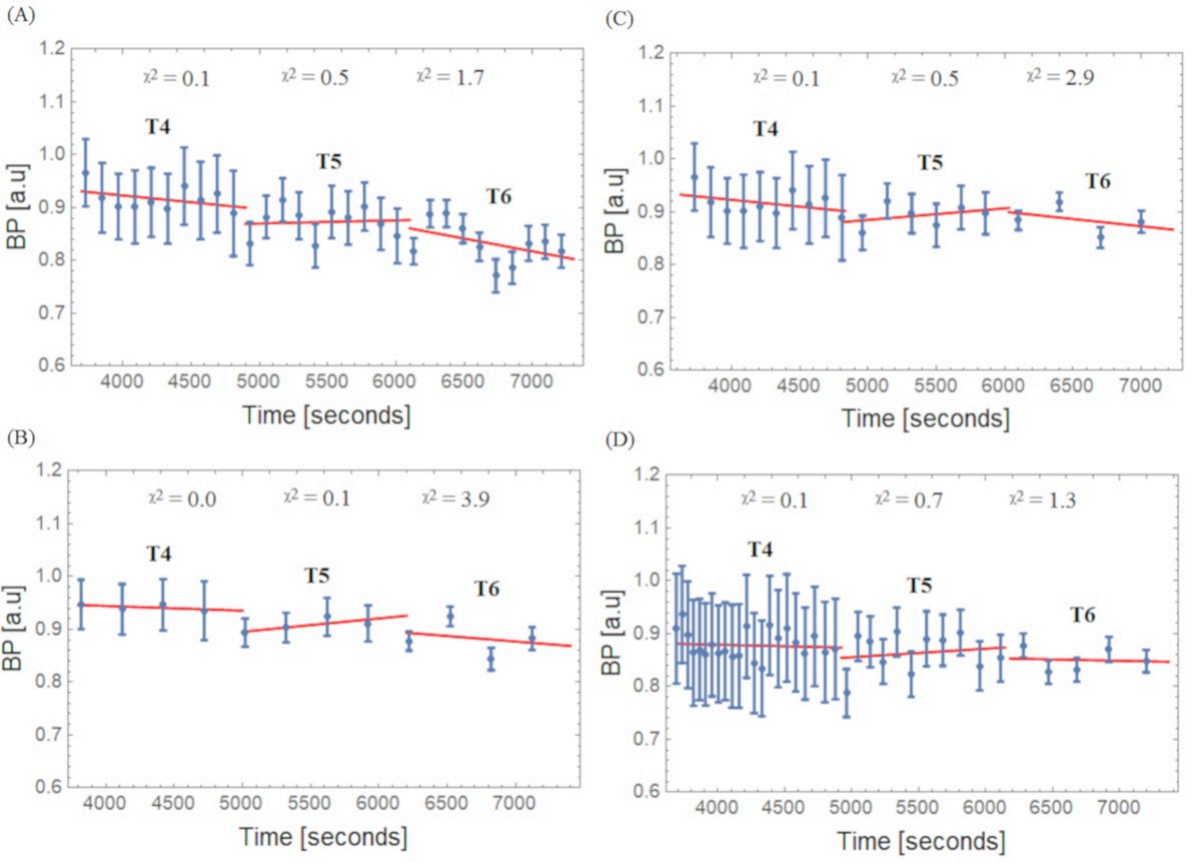
BPs and linear fits for the Hot VOI region. (A) Const 2min, (B) Const 5min, (C) Incr and (D) Const Trues framing schemes; X ^2^: Goodness of linear fit over degrees of freedom.

Fig 7 shows the BPs calculated with (3) using the REF and Hot VOIs for the four different framing schemes and their respective linear fit per interval (T4, T5, and T6). Slope values ± uncertainty for the BPs values are given in Table 2. The negative slope seen in the Hot VOI region during the low count interval T6 for all framing scheme methods becomes smallest for the Const Trues scheme. When this framing scheme was applied, the variation of the slope between T4 to T6 became minimal compared to the other framing schemes.

**Table 2:** Slope* values for BP curves.

Frame Scheme	Half-Life (T_1/2_) Interval	Slope (%/h) in Hot
Const 2 min	T4	-10.1 ± 8.5
	T5	2.4 ± 12.4
	T6	-20.9 ± 14.9
Const 5 min	T4	-3.4 ± 3.5
	T5	10.4 ± 5.4
	T6	-8.5 ± 23.9
Incr	T4	-10.1 ± 8.4
	T5	8.8 ± 13.4
	T6	-11.2 ± 20.3
Const Trues	T4	-2.4 ± 6.9
	T5	6.7 ± 13.6
	T6	-2.1 ± 14.9

*Slope–Obtained from linear fits for data presented in Fig 7, and corresponding ± uncertainty.

## Discussion

This study was performed in the context of combined PET/MRI examinations. Looking at ^11^C-labeled neuro-receptor ligands, the PET part focuses on changes of the binding potential during a dynamic acquisition with a BI protocol. During an acquisition period of three half-lives (T4, T5, and T6) of ^11^C, the recorded counts per frame decrease considerably and may cause a count dependent bias of the reconstructed activity and also of the derived binding potential. With this background, simple ratio methods were used to investigate the quantitation BP bias resulting from framing schemes previously used and from a new alternative framing scheme, known as Const Trues.

Quantitation bias as a result of low counts in PET images reconstructed with the 3D OP-OSEM algorithm has been studied by several groups and special emphasis has been placed on the impact of the bias on estimated activity concentrations and binding potentials in neuro-receptor studies [8–10, 16, 28, 34, 35]. At low count rates, the distribution of the reconstructed events per voxel is asymmetric, leading to a bias in the mean of the voxel activity concentration values [9]. MLEM and OP-OSEM reconstructions tend to be biased in regions with low activity concentrations, particularly if these regions are surrounded by regions of high activity concentrations [16]. Slambrouck et al. also demonstrated that regions with low activity concentrations will converge much more slowly and a very high number of iterations would be required to eliminate the positive bias [16]. As the image noise increases with an increasing number of iterations, it is difficult to set the number of iterations high enough to avoid this convergence bias. Moreover, since there are other sources of bias apart from iteration numbers, a completely bias-free image is not possible [16] and at low count statistical levels, other factors, such as random estimation, can be critical. This is because single rates are not constant and/or the activity distribution is not static during the frame, thus motivating our approach of carefully choosing the framing for the iterative image reconstruction. Previous works have reduced the random events estimation bias considerably by applying a reduction variance given by VRR algorithms [9, 14, 28]. However, other factors, such as scatter overcorrection, global dead time correction instead of block-wise correction, inconsistencies in the attenuation map, etc., can still contribute to the reconstruction bias. These factors are not within the scope of this work and this question needs further investigation.

Other methods to reduce bias at low count rates have been proposed, e.g., Hong et al. [31] used a method called complementary frame reconstruction. This method involves the indirect formation of a low count image with very short time frames through the subtraction of two frames with longer acquisition times (high statistics). The short time frame is then excluded from the second, long frame data prior to reconstruction. The method was tested with a phantom and with clinical data using HRRT and Biograph mCT scanners and with OP-OSEM reconstruction. In contrast to this study, the authors focused their work on applications relating to the estimation of the arterial input function, which requires very short time frames. While the method from Hong et al. is not limited to such short frames, they comment that random correction factors should be similar in order to avoid quantification mismatch. In this work, we consider acquisition intervals between one and three half-lives of ^11^C, therefore this condition is not fulfilled. Our alternative Const True framing scheme aims to minimize the bias change in a sequence of reconstructed images from a dynamic PET scan. The goal is to maximize the possibility of identifying pre- and post-task effects together with other modalities and during the same brain conditions. In addition, by increasing the possibility of detecting binding differences by reducing the bias and keeping it at the same level during the pre- and post-task periods, direct comparison is feasible. Since even when using 3D OP-OSEM + VRR reconstructions the bias cannot be avoided entirely, we hypothesize that, if the bias is mainly due to the low count rates, it can be mitigated and maintained at a constant level by keeping the counts per frame constant during the entire PET acquisition interval, i.e. keeping bias propagated into BP values constant during the entire acquisition interval for the analysis.

In recent years, new algorithms have been proposed to improve image quality by reducing noise, improving resolution through resolution models or including time-of-flight (ToF) technology mostly for lesion detectability purposes [19]. To the best of our knowledge, none of the studies that have investigated the new reconstruction algorithms have also included an analysis that studies their impact on reconstruction bias at low counts with the simple ratio approaches for BP analysis as proposed in our study with dynamic PET acquisitions. Furthermore, as the BrainPET insert used in this study is not ToF capable we do not have reconstruction algorithms at our disposal to compare with our current analysis.

The findings of our study support a previous bias investigation undertaken by Planeta-Wilson and colleagues using HRRT and OP-OSEM (MOLAR) reconstruction. Here, a bias of -4±2% was shown for GM (high activity concentration region) and 4±5% for white matter (low activity concentration) regions [7]. In terms of bias range, for low and high activity concentration regions, our results showed agreement, since the bias range values were closer for REF with 2.1±0.8% and Hot with 2.9±1.8% with respect to activity concentration accuracy (with Const Trues framing). However, in our case, the mean bias values were positive for both regions. Note that the differences of around 2% are probably due to our higher PET sensitivity of 6% [36], compared to 3% of the HRRT system [37]. In addition, based on data obtained from the fit analysis (slope changes), we also observed a negative bias for high activity concentration regions when the frame length was shortened (low counts). Our study differs from previous studies reporting on the analysis approach since we additionally evaluated the slope of bias changes from a complete dynamic PET acquisition rather than the bias for single frames from static PET acquisitions but with different count statistics. Furthermore, our results completely differ from others in terms of the amount of bias found. Johansson et al., reported a significantly higher bias, which was in the range of -16% to -8% in high activity concentration regions (1M and 200k counts, respectively), using HRRT and 3D OP-OSEM. Van Velden et al., reported a bias of around -9% to -14% in GM (Hoffman phantom in 5 seconds frame) using the same scanner and reconstruction [5, 28]. Moreover, Reilhac and colleagues reported a bias of up to 80% in the CER region for the end of the activity time course in simulations using 3D UW-OSEM [8].

It is important to interpret these results carefully since our study differs in some aspects, for example, in terms of activity concentration and high to low activity concentration ratios used (in this study 1:1.85 to mimic our ABP protocol), the radioisotope and radiopharmaceuticals, and the range of analyzed statistics. Moreover, there are some configuration differences between scanners (i.e. our scanner sensitivity is higher than the majority of scanners used in previous studies) and the 3D OSEM reconstruction presented (number of iterations and subsets), post-processing smoothing, etc. However, in the interest of comparability, we sought to compare our results with the closest 3D OP-OSEM image reconstructions and statistic ranges.

In terms of bias propagation into binding values, our study is in agreement with van Velden et al. [28], where a negative BP bias (-14% in the referred study) was reported when using reference tissue models. In our phantom study, a negative BP bias was also found, and the slope of the bias change was up to -20.9±14.9% per hour (Const 2 min). When mitigated with the Const Trues framing scheme, the slope of the bias change was -2.1±14.9% per hour in the Hot VOI region (see in Table 2, T6). It is important to emphasize that our approach is in the context of BI studies. The BP values in some of the other studies were obtained mainly from bolus only protocols and kinetic modeling instead of simple ratio methods. Since count dependent changes in quantitation bias are introduced during the iterative image reconstruction process, parameters estimated via kinetic modeling will potentially be affected as well. Based on that, we hypothesize, that pure bolus studies using ^11^C radioligands may suffer from bias issues similar to those found in our study and it might be possible to mitigate the bias with the alternative Const Trues framing approach. However, further studies for evaluating this approach are needed.

Evaluating the slope of the bias change was chosen as the approach in this study because we expected a similar bias between time intervals (T4, T5, and T6) and along the BPs when applying the Const Trues framing scheme. In this case, the slope should tend to zero and should not significantly change from interval to interval. At the same time, the evaluation of the slope represents a good first-order approximation for changes in the bias for all framing schemes.

It should also be noted that the negative slope change of bias in the Hot VOI region and the positive slope change of bias in the REF VOI region leads to an amplified underestimation of BP (3) by the simple ratio method in the T6 time interval. This is an important point of consideration for neuro-receptor studies using equilibrium conditions, especially if the aim of the study is to evaluate the BP prior, during, and post a specific task. This holds especially for cognitive tasks where the potentially induced effects are caused by endogenous release change and are thus expected to be smaller than pharmacological challenges, e.g., video game playing tasks with [^11^C]Raclopride, 13% BP decrease versus psychostimulants 10% to 20% BP decrease [38]. In such a case, the bias could mask or alter the measured effect in regions that are similar in counts and activity concentration ratio to the one presented in this study. This is because the BP would be around 10.8% during the time interval T4 and 3.8% during the time interval T6 when a scheme with Const frames is used (see in Fig 5). This trend was also observed in an example with ABP in-vivo TACs and BP values (see in S1 and S2 Figs in S1 File). We observed a relative change of up to -20.9±14.9% per hour in T6 in the Hot VOI region with the Const 2 min framing scheme, -8.5±23.9% with the Const 5 min framing scheme, -11.2±20.3% with the Incr framing scheme, and a mitigated bias of up to -2.1±14.9% with the Const Trues framing scheme. These purely bias induced changes could be wrongly attributed to changes in the BP caused by the task and can give rise to misinterpretation and incorrect conclusions, especially when Cont framing schemes are used. With the proposed, Const Trues framing scheme, the average BP bias could be reduced/mitigated to mean values lower than 5% (2.56±3.92%) in the phantom study (see Fig 5) and to values which are nearly constant for time intervals T4, T5, and T6. A drawback of the proposed framing method is the resulting very short time frames at the beginning of the acquisition, which are a consequence of the reference counts number in the last frame. This also leads to an increased variability (Fig 5). Nevertheless, a period of time is required to reach equilibrium in neuro-receptor studies, e.g., for ABP this usually starts at around 30 minutes p.i., so this drawback is tolerable for our study since the relevant information in this multimodal PET/MR study is obtained during the equilibrium phase interval of the acquisition.

For the Incr framing, a reduction in the mean BP bias values was also observed (5.63±2.85%). However, in relation to the difference between time intervals T4, T5, and T6, a higher bias (around 7.8%) can be seen in T4 for Incr schemes compared to Const Trues (lower than 5% from T4 to T6), thus the Incr framing scheme is suboptimal with respect to bias stability. According to our findings, framing schemes with constant frame lengths are less suitable for the evaluation of BP when used for the analysis of tasks induced effects in neuro-receptor studies as the bias range caused by these schemes has high variability between the time intervals (from T4 = 10.8% to T6 = 3.8%). In Figs 3 and 4, the falling (T4 and T5) and rising (T6) trends of the bias for the REF VOI region and the falling behavior of the bias for the Hot VOI region can be clearly observed and is particularly evident with constant framing schemes. Similar results were found for the TACs from a human brain study example with ABP (especially the rising behavior in the last time interval for CER and falling behavior for the anterior cingulate cortex (ACC), see S1 and S2 Figs in S1 File). This occurrence was also observed by van Velden et al. [28] using the pons as a reference region in a study with [^11^C]Flumazenil.

The differences observed, most notably for constant framing schemes, from T4 to T5 (see Figs 3 and 4) are in line with the findings of van Velden et al. [28], where an increasing ratio between reconstructed and true activity was related to an increasing noise equivalent count rate (NEC) (see Fig 4C in the referred publication). The activity concentration range and activity ratio range used in this analysis results in a range of 300 kcps to 30 kcps prompt counts. In this range of observed prompt count rates, the changes of noise equivalent counts of the BrainPET insert are significant. The constant counts framing scheme used in this study was not based on the NEC, since it is not accessible prior to image reconstruction with scatter correction. Consequently, the true prompt coincidence rated reduced by the estimated random coincidence rate was employed as it is readily available prior to image reconstruction. Please note, that in this work, the true coincidence rate is understood as the difference between the prompt coincidence rate and the estimated random coincidence rate.

In summary, all evaluated framing schemes, even with the minimized bias range achieved with our proposed Const Trues framing scheme, cause a bias of at least 2.56±3.92%, which should be taken into account for the conclusions drawn from the observed BP values. Moreover, when task effects are evaluated with the Const Trues framing scheme, the task must induce a change in the BP of at least 2.56±3.92% in order to be observable in equilibrium studies with ABP during the analyzed time interval. Conventional, non-constant framing schemes have been commonly used in order to adjust the sampling of TACs to the rate of its variation (e.g. bolus phase, washout phase, and equilibrium phase). Our proposed approach, however, is motivated very differently, since it aims to adjust the frame lengths in order to keep the bias of the BP as constant as possible in the interval of interest, thus enabling a valid comparison of effects prior to, during, and posterior to the task and avoiding misinterpretations of possible effects. The reduction and stabilization of the bias over a longer scan interval (in this case 60–65 minutes, which will lead to varying bias with conventional framing) would be very relevant for evaluating changes in the BP during a cognitive task since the effect of tracer displacement owing to endogenous neurotransmitter release is expected to be small and therefore easily masked by a changing reconstruction bias.

The Const Trues framing scheme method presented here opens new possibilities for clinical equilibrium studies under BI protocols by reducing and maintaining the BP bias constant within subjects and during cognitive tasks but can also be extended to reduce the impact of BP bias for comparisons between subjects and during cognitive tasks. It enables new possibilities as the potential detection of small changes in BP induced by cognitive tasks for healthy or diseased groups, since the spurious variation due to BP bias within and between subjects is reduced.

Here we have included representative framing schemes (similarly reported for bolus and BI protocols with ABP) alongside conventional stepwise adaptation of the frame length to the change rates of the TACs of the ^11^C-labeled radiotracer. Regularized iterative algorithms and the inclusion of resolution modeling [9] have been reported to potentially affect the quantitation bias in addition to reducing the variance and image noise. However, to the best of our knowledge, the amount of bias low count rate and its propagation into BP values and the impact of the framing scheme has still not been analyzed in detail for regularized reconstruction algorithms and algorithms with resolution modeling. In the context of repeated whole-body FDG-PET for therapy monitoring, Karaoglanis et al. [39] evaluated the bias for low statistics in simulated thoracic lesions using regularized OSEM with noise suppression and concluded that the bias did not differ significantly when compared to the post-filtered OSEM bias values were up to 7%, but increasing bias for stronger regularization was also reported.

From our understanding, since determining the BP is widely used in neuro-receptor studies and no special condition must be fulfilled to apply the Const True count framing scheme together with the BP ratio method, the Const True count framing scheme has general applicability. Moreover, the Const Trues framing scheme is instrument independent as PET imaging is based on reconstructing images from the spatial distribution and the amount of detected true counts (true events curves are always available for all PET systems).

In light of our results and those of Jian et al. [10], who have identified scatter correction as another potential bias source, further studies are planned to investigate the influence of scatter correction at low count rates.

## Conclusion

This work aimed to analyze the change of count dependent bias in activity concentrations estimated from dynamic PET acquisitions and its propagation into binding potential values for equilibrium studies. This was achieved using an ^11^C filled decay phantom study with the same conditions as an ABP study protocol. We proposed a Const Trues framing scheme, which resulted in minimal bias values and minimal change of the bias when compared to several conventional framing schemes. The minimized bias can be understood as a lower limit for the observability of any potentially endogenous response to cognitive tasks in neuro-receptor studies with ABP using the BI protocol. For our study configuration with the proposed Const Trues framing scheme, this lower limit was specified as 2.56±3.92%. Our approach uses fundamental principles of PET imaging and BP evaluation and is therefore generally applicable to dynamic, quantitative PET acquisitions. Further studies are required to estimate the bias introduced by other sources, such as scatter correction, and to study the effect of image reconstruction regularization methods and resolution modeling.

## Supporting information

S1 File (DOCX)
