# Supplementary material for: Bias evaluation and reduction in 3D OP-OSEM reconstruction in dynamic equilibrium PET studies with 11C-labeled for binding potential analysis
Source: PLoS One. 2021 Jan 22;16(1):e0245580. doi: 10.1371/journal.pone.0245580 (PMC7822533; doi:10.1371/journal.pone.0245580)
Supplement: S1 File — (DOCX) [file pone.0245580.s001.docx]

The proposed framing scheme (Const Trues) was applied to a data set from a healthy volunteer from the ABP study as an example and compared with other framing schemes (Const and Incr). TACs and BP_ND_ (BP was changed to BP_ND_ for human data, but both were calculated based on simple ratio methods) plots are shown in Figs 1 and 2 and Tables 1 and 2, respectively. The fit results were obtained during the equilibrium from 1800 seconds to the acquisition end (close to T5 and T6 range in the phantom measurement).


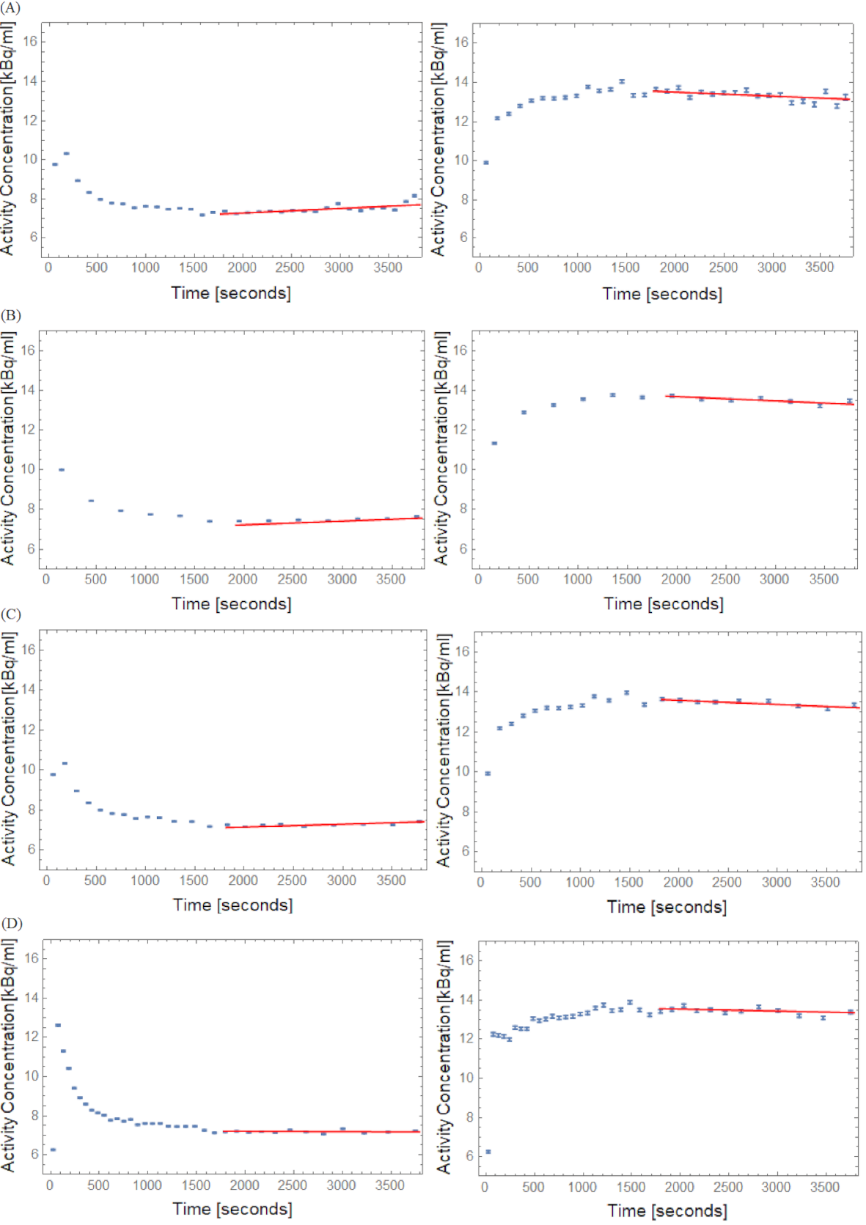


**S1 Fig.** **TAC and linear fits during equilibrium from a human brain study.** The CER (left side) and ACC (right side) regions for (A) Const 2 min, (B) Const 5 min, (C) Incr and (D) Const Trues framing schemes.

**
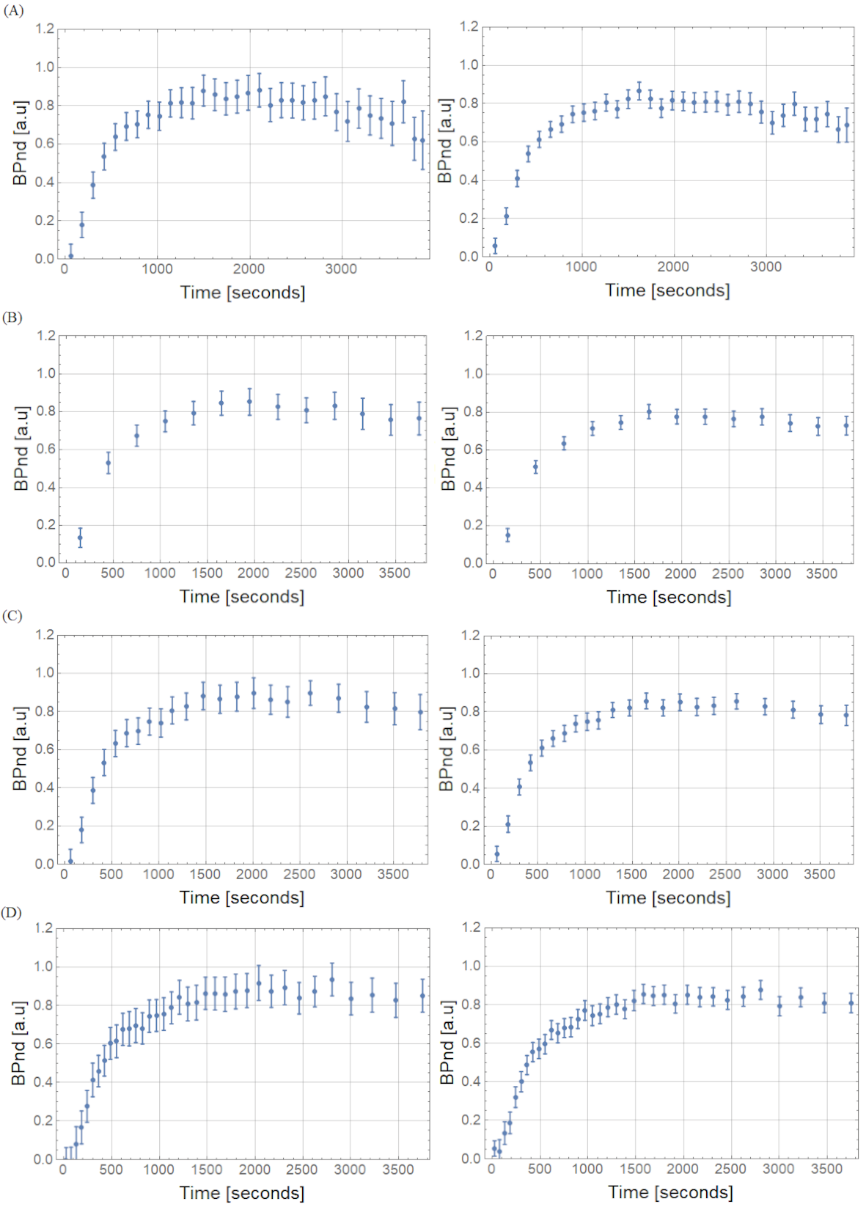
**

**S2 Fig.** **BP_ND_ ± SE values from a human brain study.** The ACC (left side) and posterior temporal cortex (Post-Tl) (right side) regions for (A) Const 2 min, (B) Const 5 min, (C) Incr and (D) Const Trues framing schemes.

**S1 Table. Slope* values for time activity curves – Human data.**

| **Frame Scheme** | **Slope (%/h) in CER** | **Slope (%/h) in ACC** |
| --- | --- | --- |
| Const 2 min | 12.3 ± 2.8 | -7.7 ± 2.0 |
| Const 5 min | 5.3 ± 1.0 | -4.7 ± 1.7 |
| Incr | 3.4 ± 1.6 | -5.1 ± 1.1 |
| Const Trues | 0.6 ± 1.9 | -4.9 ± 1.9 |

*Slope – Obtained from linear fits for data presented in S1 Fig, and corresponding ± uncertainty.

**S2 Table. Slopes* values for BP_ND_ curves – Human data.**

| **Frame Scheme** | **Slope (%/h) in ACC** | **Slope (%/h) in Post-Tl** |
| --- | --- | --- |
| Const 2 min | -39.3 ± 6.4 | -29.8 ± 4.3 |
| Const 5 min | -20.1 ± 3.1 | -16.5 ± 2.2 |
| Incr 2-3-5 min | -15.2 ± 3.9 | -12.8 ± 3.0 |
| Const Trues | -7.7 ± 5.0 | -6.1 ± 3.9 |

*Slope – Obtained from linear fits for data presented in S2 Fig, and corresponding ± uncertainty.
